# Acquired Thrombotic Thrombocytopenic Purpura Following Inactivated COVID-19 Vaccines: Two Case Reports and a Short Literature Review

**DOI:** 10.3390/vaccines10071012

**Published:** 2022-06-24

**Authors:** Imen Ben Saida, Iyed Maatouk, Radhouane Toumi, Emna Bouslama, Hajer Ben Ismail, Chaker Ben Salem, Mohamed Boussarsar

**Affiliations:** 1 Medical Intensive Care Unit, Faculty of Medicine of Sousse, University of Sousse, Sousse 4000, Tunisia; imen.bensaida@yahoo.com (I.B.S.); maatouk.yed@gmail.com (I.M.); radhouane.toumi@gmail.com (R.T.); 2 Research Laboratory Heart Failure, LR12SP09, Farhat Hached University Hospital, University of Sousse, Sousse 4000, Tunisia; 3 Department of Hematology, Faculty of Medicine of Sousse, University of Sousse, Sousse 4000, Tunisia; emna.bouslama@hotmail.com (E.B.); hajerbenismail68@gmail.com (H.B.I.); 4 Department of Pharmacovigilance, Faculty of Medicine of Sousse, University of Sousse, Sousse 4000, Tunisia; bensalem.c@gmail.com

**Keywords:** vaccines, COVID-19, safety, purpura, thrombotic thrombocytopenic

## Abstract

The severe acute respiratory syndrome coronavirus 2 (SARS-CoV-2) outbreak in December 2019, causing millions of deaths all over the world, and the lack of specific treatment for severe forms of coronavirus disease 2019 (COVID-19) have led to the development of vaccines in record time, increasing the risk of vaccine safety issues. Recently, several cases of thrombotic thrombocytopenic purpura (TTP) have been reported following COVID-19 vaccination. TTP is a rare disease characterized by thrombocytopenia, microangiopathic hemolytic anemia and ischemic end-organ lesions. It can be either congenital or acquired. Various events such as viral infections, medication, pregnancy, malignancies, and vaccinations may cause TTP. Here, we report two cases of acquired TTP following Sinopharm COVID-19 vaccine (BBIBP-CorV) and Sinovac COVID-19 vaccine (CoronaVac). Diagnosis was based on clinical presentation and confirmed with a severe reduction in the activity of von Willebrand factor-cleaving protease ADAMTS-13 and the presence of inhibitory autoantibodies. The two patients were successfully treated with corticosteroids, plasma exchange therapy and rituximab in the acute phase. In the literature, the reported cases of TTP induced by COVID-19 vaccination occurred after Adenoviral Vector DNA- and SARS-CoV-2 mRNA-Based COVID-19 vaccines. To the best of our knowledge, this is the first report of acquired TTP after inactivated virus COVID-19 vaccination.

## 1. Introduction

Severe acute respiratory syndrome coronavirus 2 (SARS-CoV-2) is a novel virus first detected in Wuhan in December 2019. A few months later, the World Health Organization (WHO) declared a worldwide pandemic. This virus can cause severe viral pneumonia with acute respiratory distress syndrome causing millions of deaths [1,2]. Currently, there is no effective treatment for coronavirus disease 2019 (COVID-19) [3]. However, several vaccines have been developed worldwide to reduce COVID-19 mortality and morbidity [4]. These vaccines have obtained emergency use approval by the WHO in several countries, increasing the risk of vaccine safety issues, and some adverse events have been reported [5,6,7]. Most frequent were injection site reactions or systemic effects (e.g., fatigue, headache, body pain, fever) with rare serious adverse events (e.g., anaphylaxis, Guillain-Barré, thrombosis with thrombocytopenia Syndrome) [8,9,10]. Several cases of thrombotic thrombocytopenic purpura (TTP) induced by COVID-19 vaccination have been reported in the literature [11,12,13,14]. TTP is a rare hematologic disorder classically characterized by the pentad of fever, hemolytic anemia, thrombocytopenia, renal failure, and neurologic dysfunction. However, most patients do not have the entire pentad [15]. This disease is caused by a severe decrease in the activity of the von Willebrand factor-cleaving protease ADAMTS-13 which can be either congenital or acquired due to anti-ADAMTS-13 autoantibodies [11]. Various events may initiate the production of those antibodies such as viral infections, medication, pregnancy, malignancies and, occasionally, vaccinations [16]. Here, we report two cases of acquired TTP after two inactivated COVID-19 vaccines: BBIBP-CorV vaccine, known as the Sinopharm COVID-19 vaccine, and CoronaVac, known as the Sinovac vaccine. To the best of our knowledge, the present cases are the first reported cases of acquired TTP after inactivated virus COVID-19 vaccination.

## 2. Case Presentations

The Research and Ethics Committee of Farhat Hached University Hospital approved the publication of the retrospectively obtained and anonymized data of the two cases (ID number of the approval: CER 10-2022).

### 2.1. Case 1

A 38-year-old Caucasian, North African Maghrebian woman with no medical history presenting with dizziness and ecchymosis in her upper limbs was referred to the hematology department. The patient reported that she had received a first dose of an inactivated virus COVID-19 vaccine Sinopharm (BBIBP-CorV) twenty days before symptom onset. Laboratory findings revealed hemoglobin 6 g/dL, platelet count 6 × 10^9^/L, lactate dehydrogenase (LDH) 1074 UI/L, D-dimer 1200 µg/L, haptoglobin 0.54 g/L, creatinine 66 µmol/L, urea 20.7 mg/dL, total bilirubin 3.75 mg/dL and indirect bilirubin 2.88 mg/dL. Peripheral blood smear showed schistocytes (1 to 2%). During her hospital stay, the patient presented left hemi-body heaviness and dysarthria. A brain MRI revealed an ischemic stroke in the territory of the inferoposterior cerebellar artery. A curative anticoagulation was started. A few hours after ICU admission, the patient presented a sudden generalized tonico-clonic seizure with status epilepticus requiring her intubation. Glycemia and electrolytes were within the normal ranges. The patient was promptly given clonazepam and intravenous sodium valproate. Analgo-sedation was prolonged with remifentanyl and midazolam to achieve a Richmond Agitation Sedation Scale (RASS) [17] at −5 to control the status epilepticus and obtain patient–ventilator synchronization. The presence of thrombocytopenia, hemolytic anemia and neurological symptoms was indicative of a presumptive diagnosis of TTP. The PLASMIC score, used to identify patients with ADAMTS-13 deficiency in suspected TTP patients, was at 6 (range, 0–7) indicating a high risk of severe ADAMTS-13 deficiency < 10%. The patient was promptly treated by methylprednisolone 1000 mg daily for three consecutive days, then 1 mg/kg/day in combination with daily plasma exchange therapy (PEX).

Infectious screening tests (e.g., human immunodeficiency virus (HIV), hepatitis, SARS-CoV-2, Epstein-Barr virus, and cytomegalovirus) were negative. Autoimmunity investigations revealed severe ADAMTS-13 deficiency (6%) with positive anti ADAMTS-13 autoantibodies more than 15 U/mL (normal < 12 U/mL) confirming the diagnosis of an acquired TTP.

**Figure 1:**
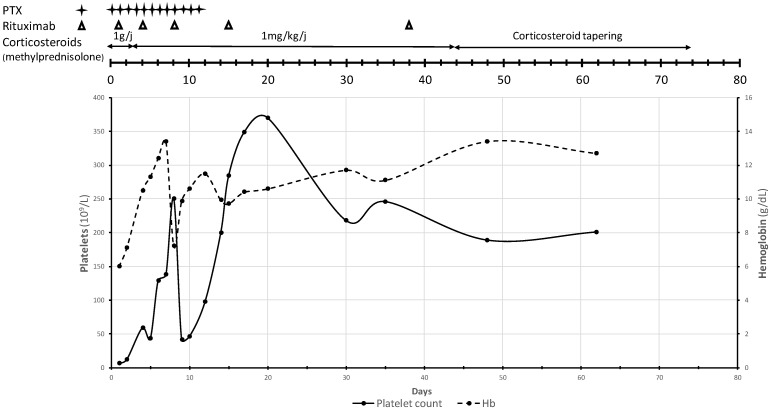
Platelet count (×10^9^/L) and hemoglobin level (g/dL) trends throughout the course of corticosteroids, plasma exchange and rituximab during the ICU stay and the follow-up period of case 1.

The patient showed clinical improvement after the third PEX with symptom resolution. She remained seizure free and was extubated on day 5 of her ICU stay. The normalization of LDH was achieved 7 days after initiation of PEX, whereas a decrease in total bilirubin to 1 mg/dL was seen on day 10 of treatment. On day 15 in the ICU, a normalization of the platelet count was observed. The patient had fully recovered after a 17-day course of glucocorticoids, 12 sessions of PEX and rituximab. Laboratory parameter improvement trends (platelet and hemoglobin level) are displayed in Figure 1. The patient was discharged with a follow up at the hematology department. Prednisone was tapered off over 5 weeks. The patient made a complete recovery and is currently living a normal life. The latest ADAMTS-13 activity at the 6-month follow-up visit showed 94%.

### 2.2. Case 2

A previously healthy 30-year-old Caucasian, North-African Maghrebian male presented to the emergency department with headache, fever, dysarthria and right hemiparesis. He had received a second dose of an inactivated COVID-19 vaccine CoronaVac, one month prior to consultation. Laboratory findings showed hemoglobin 7.2 g/dL, platelet count, 9 × 10^9^/L, LDH 1268 UI/L, D-dimer 1890 µg/L, haptoglobin 0.26 g/L and creatinine 105 µmol/L. A peripheral blood smear showed schistocytes (2%). Their PLASMIC score was at 5 (range, 0–7). A presumptive diagnosis of TTP was made. The patient was admitted to the ICU. On the initial examination, the patient had a fluctuating consciousness, dysarthria and right hemiparesis without any petechiae or purpura. The brain CT scan revealed no abnormalities. No triggering factors such as viral infections or medication, alcohol or illicit drug use were identified. Infectious screening tests including SARS-CoV-2 were negative. Investigations revealed severe ADAMTS-13 deficiency (<0.2%) with positive anti ADAMTS-13 autoantibodies (12 U/mL). All other autoimmune tests returned negative.

The patient received methylprednisolone 1000 mg daily for three consecutive days followed by prednisone 1 mg/kg/day in combination with daily PEX. Weekly infusion of rituximab for 4 weeks was started two weeks after admission due to issues concerning the patient’s health insurance.

**Figure 2:**
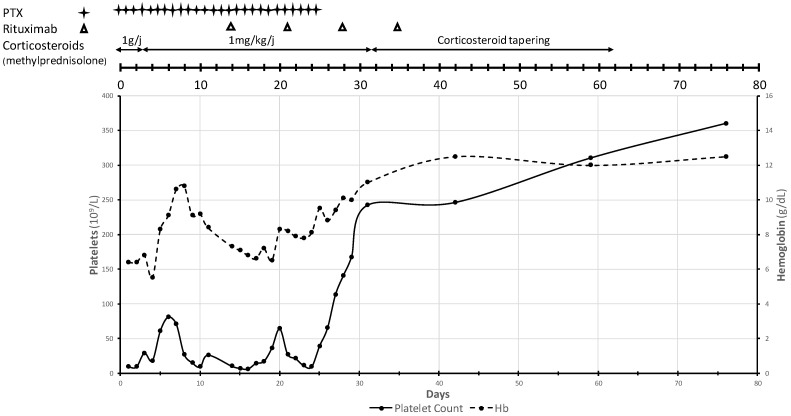
Platelet count (×10^9^/L) and hemoglobin level (g/dL) trends throughout the course of corticosteroids, plasma exchange and rituximab during the ICU stay and the follow-up period of case 2.

Neurological symptoms resolved gradually after the sixth PEX. However, there was no improvement in platelet count and LDH values, leading to prolongation of PEX therapy in association with rituximab. The laboratory findings showed a complete and sustained response at day 28 of ICU stay. The patient had fully recovered after a 31-day course, which included 26 sessions of PEX (Figure 2). The patient was discharged with hemoglobin at 10 g/dL and platelets at 180 × 10^9^/L with a follow-up at the hematology department. The steroid dose was tapered off over 4 weeks. One month later, the control of activity of ADAMTS-13 was 74%.

## 3. Discussion

Thrombotic thrombocytopenic purpura (TTP) is a rare blood disorder with an incidence of 3 to 10 cases per million adults per year [16]. It was first described by Eli Moschcowitz in 1924 [18,19]. The pathogenesis of this disorder includes the formation of small-vessel platelet rich thrombi leading to ischemic end organ injury [20]. The historical pentad (fever, hemolytic anemia, thrombocytopenia, neurological or renal dysfunction) is only seen in <10% of the patients [21,22]. Microangiopathic hemolytic anemia (reduced Hb and haptoglobin, increased LDH and presence of schistocytes) and thrombocytopenia are sufficient for presumptive diagnosis of TTP. The PLASMIC score derived by Bendapudi et al. [23] stratifies patients according to their risk of having severe ADAMTS-13 deficiency. When dichotomized at high (score 6–7) vs. low–intermediate risk (score 0–5), the PLASMIC score predicted severe ADAMTS-13 deficiency with positive predictive value at 72%, negative predictive value at 98%, sensitivity 90%, and specificity 92% [24]. A severe reduction in the activity of von Willebrand factor (VWF) cleaving metalloprotease (ADAMTS-13) (less than 10%) and the presence of inhibitory antibodies confirm the diagnosis [20].

TTP can be classified into two types: congenital or acquired (autoimmune TTP). Autoimmune TTP can be triggered by infections, malignancy, pregnancy, medications and vaccines [11,19]. Rarely, some vaccines (e.g., influenza, pneumococcus, rabies and H1N1) have been reported to induce acquired TTP [11,14,21,25,26,27]. Vaccines have been hypothesized to activate the immune system leading to autoantibody formation and hence the development of autoimmune disorders such as TTP [21,28].

Worldwide, in response to the COVID-19 pandemic, several vaccines have been developed using various techniques: messenger RNA (mRNA) (Pfizer-BioNTech [BNT162b2], Moderna and CureVac), human or primate adenovirus vectors (Janssen-Johnson & Johnson [Ad26.COV2-S], Astra-Zeneca [chAdOx1 nCoV-19], Sputnik-V, and CanSino) and an inactivated whole-virus SARS-CoV-2 (Bharat Biotech, Sinopharm and Sinovac) [22]. The emergency use authorization of these vaccines in several countries increased the risk of safety issues [5,7]. In the literature, there have been some reported cases of TTP following Adenoviral Vector DNA- and SARS-CoV-2 mRNA-based COVID-19 vaccines [11,29]. Indeed, vaccines against viral pathogens have been reported to be associated with onset and/or relapse of TTP [30]. This rare autoimmune disease may occur after the first or the second dose of COVID-19 vaccines, typically one to two weeks after vaccination [13].

For TTP, vaccine-induced immune thrombotic thrombocytopenia (VITT) is a differential diagnosis. VITT is another adverse event that has been recently reported after COVID-19 vaccination. It is a novel clinical syndrome demonstrating striking similarities to TTP. VITT is diagnosed clinically by the presence of mild to severe thrombocytopenia, documented evidence or suspicion of thrombosis and positive antibodies against platelet factor 4 (PF4) [31,32,33]. In the present two cases, severely reduced ADAMTS-13 activity and the presence of schistocytes or microangiopathic hemolytic anemia on the blood smear support the diagnosis of TTP. Temporal association and absence of other triggering factors for secondary TTP led to the diagnosis that this disorder was induced by COVID-19 vaccination. The mechanism linking TTP with COVID-19 vaccines is poorly understood [12]. However, it has been well established that, in patients with acquired TTP, deficiency of ADAMTS-13 results from autoimmune inhibitors of the ADAMTS-13 protease. The levels of the ADAMTS-13 inhibitors tend to be low (<10 U/mL), often receding to even lower or undetectable levels within weeks or months. Such characteristics of the ADAMTS-13 inhibitors suggest that the immune response is induced by exposure to exogenous antigens with molecular mimicry to ADAMTS-13 [34]. The two cases were recorded within a two-year-long COVID-19 pandemic; including just one year of active vaccination in Tunisia, in which more than five hundred COVID-19 patients were admitted to a 12-bed medical ICU, along with another 600 non-COVID-19 patients in the same two-year period. This highlights the scarcity of such complications in our hospital.

On 5 April 2022, a personal literature review based on a 2020–2022 PubMed search (key items: “Thrombotic thrombocytopenic purpura” AND “COVID-19 vaccines” AND “case report”) found 19 papers including 32 cases published in English language. Among these studies, TTP was reported as an adverse event of, respectively, Pfizer-BioNTech (*n* = 24), Moderna (*n* = 3), Astra-Zeneca (*n* = 4) and Janssen-Johnson & Johnson (*n* = 1) (Table 1; Results of the 32 Cases, Published During the 2020–2022 Period, Including Thrombotic Thrombocytopenic Purpura following COVID-19 Vaccination) [11,12,13,14,20,21,22,25,26,28,29,30,35,36,37,38,39,40,41].

The strength of the present study is that this is the first report of acquired TTP after inactivated virus COVID-19 vaccination. Vaccination status, vaccine name and date of doses were verified by checking the patients’ vaccination certificate in the national register of vaccination (Government’s EVAX website). In the current cases, TTP occurred 20 days after the first dose of Sinopharm and 30 days after second dose of CoronaVac. The two cases were reported to the regional pharmacovigilance center.

The present study has some limitations. First, it is a case report of only two patients. Second, addressing the question of possible prior SARS-CoV-2 infection was very difficult to prove definitely, unless checking for seroconversion, which could also result from the vaccine. In the present two cases, the causality relationship between the TTP and the vaccine was made very probable on a bundle of anamnestic, clinical and laboratory arguments and the chronology between vaccination and the onset of symptoms. Third, the short review was not a systematic one and used only one database.

Healthcare workers involved in COVID-19 vaccination programs need to educate the recipients of the COVID-19 vaccines about the possible adverse events. Careful clinical auto-surveillance must be conducted in the post-vaccine period. There are currently no recommended screenings for TTP when a patient has no signs or symptoms. However, clinicians should consider the possibility of TTP when evaluating thrombocytopenia following vaccination. Without the prompt initiation of adequate treatment, TTP is a life-threatening thrombotic microangiopathy. It is a medical emergency requiring rapid diagnosis and treatment, usually in intensive care units. According to the International Society of Thrombosis and Haemostasis, PEX represents the cornerstone of TTP treatment with strong recommendation for adding corticosteroids [28,42]. Rituximab (a monoclonal anti-CD20 antibody) and Caplacizumab (an anti-VWF antibody fragment) can improve TTP outcomes and decrease the duration of PEX. Caplacizumab is not yet available worldwide, and it has a significant cost [43].

## 4. Conclusions

This report highlights potential safety issues that can be encountered after COVID-19 vaccination. The benefits of vaccination in fighting the ongoing pandemic outweigh the risk of side effects. Additional surveillance is required in the post-vaccine period to detect adverse events in a timely fashion. TTP is a very rare life-threatening complication of COVID-19 vaccination. It is a medical emergency that is almost always fatal if adequate treatment is not initiated early. Further research should be conducted to correctly identify the mechanism linking thrombotic microangiopathic disorders with COVID-19 vaccines.

## Figures and Tables

**Table 1:** Results of the 32 Cases, Published During the 2020–2022 Period, Including Thrombotic Thrombocytopenic Purpura (TTP) following COVID-19 Vaccination.

Authors and Ref	Country (Year)	Old Gender	Underlying Disease	First Episode	Symptoms	Vaccine	Biology	ADAMTS 13 Activity	Treatments	Outcome
				Relapse		Dose		Autoantibody *		
						Time after Vaccination				
Chamarti et al. [20]	USA (2021)	80	Hypertension	First	Generalized weakness	Pfizer- BioNTech	Hemoglobin, 4.8 g/dL	<2%	Plasma Exchange Steroids	Improved
		Male	Diabetes		Malaise	Second dose	Platelets, 48 × 10^9^/L	182 U/mL	Rituximab	
			Hyperlipidemia			14 days	Schistocytes, +++			
			Gout				Creatinine, 212.16 µmol/L			
			Iron deficiency				LDH, 1118 UI/L			
			Anemia				Haptoglobin, <10 mg/dL			
Giuffrida et al. [14]	Italy (2021)	83	Undifferentiated connective tissue disease	First	Severe anemia	Pfizer- BioNTech	Hemoglobin, 6.1 g/dL	<10%	Plasma Exchange Steroids	Death (probably due to a
		Female	Diabetes		Macro-hematuria	First dose	Retic, 28%	40 U/mL	Caplacizumab	sudden cardiovascular event)
					Diffuse petechiae	7 days	Platelets, 46 × 10^9^/L			
							Schistocytes, 10%			
							Creatinine, 77.79 µmol/L			
							LDH, 1905 UI/L			
							Haptoglobin, <7 mg/dL			
		30	Beta-thalassemia	First	Diffuse petechiae	Pfizer- BioNTech	Hemoglobin, 8.9 g/dL	<10%	Plasma Exchange Steroids	Improved
		Female			Intense headache	First dose	Retic, 29%	77.6 U/mL	Caplacizumab	
					Fatigue	18 days	Platelets, 11 × 10^9^/L			
							Schistocytes, 5–10%			
							Creatinine, 79.56 µmol/L			
							LDH, 900 UI/L			
							Haptoglobin, <7 mg/dL			
Karabulut et al. [11]	USA (2021)	48	No	First	Acute-onset, transient right-sided weakness	Moderna Biotech	Hemoglobin, 8.8 g/dL	<3%	Plasma Exchange Steroids	Improved
		Male			Slurred speech lasting	First dose	Platelets, 10 × 10^9^/L	6.6 BEU	Rituximab	
						5 days	Schistocytes, 2–3%			
							Creatinine, 83.98 µmol/L			
							LDH, 884 UI/L			
							Haptoglobin, <10 mg/dL			
Lee et al. [28]	UK (2021)	50	Hypertension	First	Dysphasia	AstraZeneca	Hemoglobin, 9.9 g/dL	0%	Plasma Exchange Steroids	Improved
		Female			Acute upper limb numbness	First dose	Retic, 6.9%	94.93 U/mL	Rituximab	
						12 days	Platelets, 33 × 10^9^/L			
							Schistocytes, +			
							LDH, 359 UI/L			
Maayan et al. [29]	Israel (2021)	40	No	First	Somnolence	Pfizer- BioNTech	Hemoglobin, 9.9 g/dL	0%	Plasma Exchange	Improved
		Female			Fever	Second dose	Platelets, 12 × 10^9^/L	51 U/mL	Steroids	
					Macroscopic hematuria	8 days	Schistocytes, 6%		Caplacizumab	
							Creatinine, 81.35 µmol/L			
							LDH, 7129 UI/L			
		28	Morbid obesity	First	Dysarthria	Pfizer- BioNTech	Hemoglobin, 9.1 g/dL	0%	Plasma Exchange Steroids	Improved
		Male				Second dose	Platelets, 38 × 10^9^/L	113 U/mL	Caplacizumab Rituximab	
						28 days	Schistocytes, 6%			
							Creatinine, 132.63 µmol/L			
							LDH, 3063 UI/L			
		31	TTP	Relapse	Vaginal bleeding	Pfizer- BioNTech	Hemoglobin, 7.7 g/dL	0%	Plasma Exchange Steroids	Continu caplacizumab
		Female			Purpura	First dose	Platelets, 17 × 10^9^/L	64 U/mL	Caplacizumab	
						13 days	Schistocytes, 10%		Rituximab	
							Creatinine, 106 µmol/L			
							LDH, 4000 UI/L			
		30	TTP	Relapse	Purpura	Pfizer- BioNTech	Hemoglobin, 8.3 g/dL	0%	Plasma Exchange Steroids	Improved
		Male				Second dose	Retic, 8%	21 U/mL	Caplacizumab	
						8 days	Platelets, 14 × 10^9^/L		Rituximab	
							Schistocytes, 14%			
							Renal function, normal			
							LDH, 1138 UI/L			
Osmanodja et al. [35]	Germany (2021)	25	No	First	Persisting malaise	Moderna Biotech	Hemoglobin, 7.4 g/dL	<5%	Plasma Exchange Steroids	Continu caplacizumab
		Male			Fever	First dose	Retic, 233.1 10^9^/L	72.2 U/ml	Caplacizumab	
					Headache	13 days	Platelets, 29 × 10^9^/L		Rituximab	
					Word-finding difficulties		Schistocytes, 2.1%			
					Nausea, vomiting		Creatinine, 132.6 µmol/L			
					Petechial bleeding		LDH, 999 UI/L			
					Hematuria		Haptoglobin, <8 mg/dL			
Pavenski et al. [30]	Canada (2021)	84	TTP	Relapse	Lethargy	Pfizer- BioNTech	Hemoglobin, 7.2 g/dL	<1%	Plasma Exchange	Improved
		Male	Prostate cancer Hypertension Diabetes		Myalgias	First dose	Retic, elevated	>15 U/mL	Steroids	
			Gout		Anorexia	7 days	Platelets, 58 × 10^9^/L		Rituximab	
			Hypercholesterolemia				Schistocytes, +			
							Creatinine, 77 µmol/L			
							LDH, 594 UI/L			
Sissa et al. [36]	Italy (2021)	48	TTP	Relapse	Ecchymosis	Pfizer- BioNTech	Hemoglobin, 11.5 g/dL	<3%	Plasma Exchange	Improved
		Female				Second dose	Platelets, 94 × 10^9^/L	88 U/mL	Steroids	
						6 days	Schistocytes, 10%			
							Renal function, normal			
							LDH, 637 UI/L			
Waqar et al. [22]	USA (2021)	69	Hypertension Chronic kidney disease	First	Severe fatigue	Pfizer- BioNTech	Hemoglobin, 9.3 g/dL	2%	Plasma Exchange Steroids	Improved
		Male	HIV		Shortness of breath	Second dose	Retic, 2.8%	>90 U/mL	Rituximab	
			Chronic hepatitis B			7 days	Platelets, 22 × 10^9^/L			
			Deep				Schistocytes, ++			
			vein thrombosis				Creatinine, 177.68 µmol/L			
							LDH, 1229 UI/L			
Yucum et al. [37]	USA (2021)	62	Hypertension	first	Acute onset of altered mental status	Johnson and Johnson	Hemoglobin, 8.2 g/dL	<12%	Plasma Exchange Steroids	Improved
		Female	Hyperlipidemia			First dose	Retic, 8%	NA	Hemodialysis	
			Hypothyroidism			37 days	Platelets, 11 × 10^9^/L			
							Creatinine, 530 µmol/L			
							LDH, >2500 UI/L			
							ASAT/ALAT, 982/231 U/L			
Al Ahmad et al. [21]	Kuwait (2021)	37	Secondary polycythemia	first	Dizziness, fatigue	AstraZeneca-Oxford	Hemoglobin, 8.3 g/dL	2.60%	Plasma Exchange Steroids	Improved
		Male			Headache	First dose	Retic, 8%	Positive	Rituximab	
					Shortness of breath	10 days	Platelets, 14 × 10^9^/L			
					Palpitation		Schistocytes, 14%			
					Dark urine and petechiae		Renal function, normal			
							LDH, 1138 UI/L			
De Bruijn et al. [25]	Belgium (2021)	38	No	First	Spontaneous	Pfizer- BioNTech	Hemoglobin, 10.5 g/dL	0%	Plasma Exchange	Improved
		Female			bruising and petechiae	First dose	Retic, 263 10^9^/L	106.8 BEU	Steroids	
						14 days	Platelets, 46 × 10^9^/L		Caplacizumab	
							Schistocytes, 3%		Rituximab	
							Creatinine, 83.98 µmol/L			
							LDH, 631 UI/L			
Alislambouli et al. [12]	USA (2022)	61	No	First	Confusion	Pfizer- BioNTech	Hemoglobin, 6.5 g/dL	<3%	Plasma Exchange	Improved
		Male			Fever	First dose	Retic, 8%	NA	Steroids	
					Headache	5 days	Platelets, 6 × 10^9^/L		Rituximab	
					Emesis		Schistocytes, 8%			
					Dark urine		LDH, 1757 UI/L			
					Leg ecchymosis		Haptoglobin, <8 mg/dL			
Deucher et al. [38]	USA (2022)	28	TTP	Relapse	Bruising on arms	Pfizer- BioNTech	Hemoglobin, 10.5 g/dL	<2.5%	Caplacizumab	Improved
		Female				First dose	Platelets, 84 × 10^9^/L	Positive	Steroids	
						5 days	Schistocytes, ++		Rituximab	
							LDH, 205 UI/L			
							Haptoglobin, undetectable			
Innao et al. [26]	Italy (2022)	33	Hodgkin Lymphoma	First	Asthenia	Pfizer- BioNTech	Hemoglobin, 6.8 g/dL	8%	Plasma Exchange	Improved
		Female	Gray Zone Lymphoma		Drowsiness	First dose	Retic, 896 × 10^9^/L	5 U/mL (not valuable due to defects in the sample)	Steroids	
					Headache	9 days	Platelets, 12 × 10^9^/L		Caplacizumab	
					Nausea		Schistocytes, 3%			
					Abdominal pain		Creatinine, 122 µmol/L			
					Lower extremity purpura		LDH, 1280 UI/L			
							Haptoglobin, <6 mg/dL			
Kirpalani et al. [39]	Japan (2022)	14	Anxiety	First	Fatigue	Pfizer- BioNTech	Hemoglobin, 6.3 g/dL	<1%	Plasma Exchange Steroids	Improved
		Female	Iron		Headache	First dose	Platelets, 10 × 10^9^/L	72 U/mL	Caplacizumab	
			Deficiency		Confusion	14 days	Schistocytes, +		Rituximab	
					Bruising		LDH, 626 UI/L			
							Haptoglobin, <10 mg/dL			
Ruhe et al. [40]	Germany (2022)	84	No	First	Partial hemiplegia	Pfizer- BioNTech	Hemoglobin, 7.9 g/dL	1.60%	Plasma Exchange Steroids	Improved
		Female			Scattered petechiae	First dose	Platelets, 45 × 10^9^/L	82.2 U/mL	Rituximab	
						16 days	Schistocytes, 4.2%			
							Creatinine, 172.38 µmol/L			
							Haptoglobin, <10 mg/dL			
Yoshida et al. [13]	Japan (2022)	57	Acute hepatitis of unknown cause	First	Fatigue	Pfizer- BioNTech	Hemoglobin, 5.5 g/dL	<0.5%	Plasma Exchange Steroids	Improved
		Male			Loss of appetite	First dose	Retic, 496 × 10^9^/L	1.9 BU/mL	Rituximab	
					Jaundice	7 days	Platelets, 9 × 10^9^/L			
							Schistocytes, 17.6%			
							Creatinine, 138.87 µmol/L			
							LDH, 2275 UI/L			
							Haptoglobin, 3 mg/dL			
Picod et al. [41]	France (2022)	36	Systemic lupus erythematosus	First	Bruising	Pfizer- BioNTech	Hemoglobin, 10 g/dL	<5%	Plasma Exchange Steroids	Improved
		Female			Headache	First dose	Platelets, 10 × 10^9^/L	0.5 BU/mL	Rituximab	
						6 days	Schistocytes, 3%			
							Creatinine, 86.24 µmol/L			
		54	TTP	Relapse	Bruising	Moderna Biotech	Hemoglobin, 11.5 g/dL	<5%	Plasma Exchange Steroids	Improved
		Male			Diffuse	First dose	Platelets, 17 × 10^9^/L	1.1 BU/mL	Rituximab Caplacizumab	
					mucocutaneous	First dose	Schistocytes, 2%			
					bleeding	23 days	Creatinine, 149.6 µmol/L			
					Headache					
					Amnesia					
		60	TTP	Relapse	Cerebellar	Pfizer- BioNTech	Hemoglobin, 10.8 g/dL	<10%	Plasma Exchange Steroids	Improved
		Female			Syndrome	First dose	Platelets, 27 × 10^9^/L	Positive	Rituximab	
						10 days	Schistocytes, 2%			
							Creatinine, 66.88 µmol/L			
		60	No	First	Cerebellar	Pfizer- BioNTech	Hemoglobin, 6.5 g/dL	5%	Plasma Exchange Steroids	Improved
		Female			Syndrome	First dose	Platelets, 20 × 10^9^/L	52 U/mL	Caplacizumab	
					Aphasia	12 days	Schistocytes, 6%			
					Confusion		Creatinine, 80.96 µmol/L			
					Chest pain					
		38	No	First	Fever	Pfizer- BioNTech	Hemoglobin, 6.6 g/dL	<1%	Plasma Exchange Steroids	Improved
		Male			Headache	Second dose	Platelets, 9 × 10^9^/L	Positive	Rituximab Caplacizumab	
					Hemiparesis	30 days	Schistocytes, 5%			
					Bruising		Creatinine, 88.88 µmol/L			
		68	Mixed connective tissue disease	Relapse	Dizziness	Pfizer- BioNTech	Hemoglobin, 10.9 g/dL	2%	Plasma Exchange Steroids	Improved
		Male	TTP			First dose	Platelets, 39 × 10^9^/L	-	Rituximab Caplacizumab	
						17 days	Schistocytes, 1%			
							Creatinine, 69.52 µmol/L			
		66	No	First	Facial paralysis	AstraZeneca-Oxford	Hemoglobin, 7.9 g/dL	<5%	Plasma Exchange	Improved
		Male				First dose	Platelets, 11 × 10^9^/L	-	Steroids	
						8 days	Schistocytes, 4%		Rituximab	
							Creatinine, 81.84 µmol/L		Caplacizumab	
		70	Ischemic strokes	First	Coma	AstraZeneca-Oxford	Hemoglobin, 8 g/dL	11%	Intravenous Immunoglobulins Plasma Infusion Steroids	Death 2 month
		Female	Hypertension		Hemiparesis	First dose	Platelets, 6 × 10^9^/L	140 U/mL	Rituximab Caplacizumab	after presentation
						10 days	Schistocytes, 2%			
							Creatinine, 79.2 µmol/L			
		22	No	First	Coma	Pfizer- BioNTech	Hemoglobin, 6.8 g/dL	6%	Plasma Exchange Steroids	Improved
		Male			Seizures	Second dose	Platelets, 10 × 10^9^/L	Positive	Rituximab	
					Purpura	18 days	Schistocytes, 2%			
					Fever		Creatinine, 101.2 µmol/L			
		20	Systemic lupus erythematosus	First	Systemic lupus erythematosus Flare	Pfizer- BioNTech	Hemoglobin, 5.3 g/dL	<10%	Plasma Infusion Steroids	Improved
		Female			Polyarthritis	First dose	Platelets, 51 × 10^9^/L	50 U/mL		
					Erythema	25 days	Schistocytes, 3%			
							Creatinine, 88 µmol/L			

* Autoantibodies to ADAMTS-13 was assessed either as the titer of total autoantibodies with a simplified enzyme-linked immunosorbent assay (ELISA) and expressed in arbitrary units (U/mL; normal < 12 U/mL) or as the titer of inhibitory antibodies using an alternative methodology (Bethesda assay) expressed in Bethesda Units (BU/mL; normal < 1 BU/mL) or BEU (normal < 0.4). NA: Not available. Retic, reticulocytes; LDH, Lactate dehydrogenase; +++, semi-quantitative appreciation of schistocytes.

## Data Availability

The datasets used and/or analyzed during the current study are available from the corresponding author upon a reasonable request.
